# Supplementary material for: Hyperlactatemia associated with elective tumor craniotomy: Protocol for an observational study of pathophysiology and clinical implications
Source: PLoS One. 2022 Jul 21;17(7):e0271682. doi: 10.1371/journal.pone.0271682 (PMC9302830; doi:10.1371/journal.pone.0271682)
Supplement: S1 File — Strobe checklist for “Hyperlactatemia associated with elective tumor craniotomy: protocol for an observational study of pathophysiology and clinical implications”. (DOC) [file pone.0271682.s001.doc]

STROBE Statement—Checklist of items that should be included in reports of ***cohort studies***

“Hyperlactatemia associated with elective tumor craniotomy: protocol for an observational study of pathophysiology and clinical implications.”

|  | Item No | Recommendation |
| --- | --- | --- |
| **Title and abstract** | 1 | (*a*) Indicate the study’s design with a commonly used term in the title or the abstract  Page: 1 Line: 1-2 |
|  |  | (*b*) Provide in the abstract an informative and balanced summary of what was done and what was found  Page: 2 Line: 19-37 |
| Introduction | | |
| Background/rationale | 2 | Explain the scientific background and rationale for the investigation being reported  Page: 3-4 Line: 40-75 |
| Objectives | 3 | State specific objectives, including any prespecified hypotheses  Page: 4-5 Line: 76-85 |
| Methods | | |
| Study design | 4 | Present key elements of study design early in the paper  Page: 5 Line: 89-90 |
| Setting | 5 | Describe the setting, locations, and relevant dates, including periods of recruitment, exposure, follow-up, and data collection  Page: 5 Line: 93-95  Page: 14 Line: 272-273 |
| Participants | 6 | (*a*) Give the eligibility criteria, and the sources and methods of selection of participants. Describe methods of follow-up  Page: 5-6 Line: 98-113  Page: 7 Line: 135-142 Figure: 1  Page: 9 Line: 176-182 |
|  |  | (*b*) For matched studies, give matching criteria and number of exposed and unexposed  Not relevant |
| Variables | 7 | Clearly define all outcomes, exposures, predictors, potential confounders, and effect modifiers. Give diagnostic criteria, if applicable  Page: 10-12 Line: 205-249 |
| Data sources/ measurement | 8* | For each variable of interest, give sources of data and details of methods of assessment (measurement). Describe comparability of assessment methods if there is more than one group  Page: 8-9 Line: 144-182 |
| Bias | 9 | Describe any efforts to address potential sources of bias  Page: 14 Line: 275-295 |
| Study size | 10 | Explain how the study size was arrived at  Page: 6-7 Line: 124-129 |
| Quantitative variables | 11 | Explain how quantitative variables were handled in the analyses. If applicable, describe which groupings were chosen and why  Page: 13 Line: 251-259 |
| Statistical methods | 12 | (*a*) Describe all statistical methods, including those used to control for confounding  Page: 13 Line: 251-259 |
|  |  | (*b*) Describe any methods used to examine subgroups and interactions (statistic)  Page: 13 Line: 251-259 |
|  |  | (*c*) Explain how missing data were addressed  Page: 14 Line: 293-295 |
|  |  | (*d*) If applicable, explain how loss to follow-up was addressed  Page: 14 Line: 293-295 |
|  |  | (*e*) Describe any sensitivity analyses  Not relevant |
| Results | | |
| Participants | 13* | (a) Report numbers of individuals at each stage of study—eg numbers potentially eligible, examined for eligibility, confirmed eligible, included in the study, completing follow-up, and analysed  Not relevant (Study protocol, no results available) |
|  |  | (b) Give reasons for non-participation at each stage  Not relevant (Study protocol, no results available) |
|  |  | (c) Consider use of a flow diagram  Not relevant (Study protocol, no results available) |
| Descriptive data | 14* | (a) Give characteristics of study participants (eg demographic, clinical, social) and information on exposures and potential confounders  Not relevant (Study protocol, no results available) |
|  |  | (b) Indicate number of participants with missing data for each variable of interest  Not relevant (Study protocol, no results available) |
|  |  | (c) Summarise follow-up time (eg, average and total amount)  Not relevant (Study protocol, no results available) |
| Outcome data | 15* | Report numbers of outcome events or summary measures over time  Not relevant (Study protocol, no results available) |
| Main results | 16 | (*a*) Give unadjusted estimates and, if applicable, confounder-adjusted estimates and their precision (eg, 95% confidence interval). Make clear which confounders were adjusted for and why they were included  Not relevant (Study protocol, no results available) |
|  |  | (*b*) Report category boundaries when continuous variables were categorized  Not relevant (Study protocol, no results available) |
|  |  | (*c*) If relevant, consider translating estimates of relative risk into absolute risk for a meaningful time period  Not relevant (Study protocol, no results available) |
| Other analyses | 17 | Report other analyses done—eg analyses of subgroups and interactions, and sensitivity analyses  Not relevant (Study protocol, no results available) |
| Discussion | | |
| Key results | 18 | Summarise key results with reference to study objectives  Not relevant (Study protocol, no results available) |
| Limitations | 19 | Discuss limitations of the study, taking into account sources of potential bias or imprecision. Discuss both direction and magnitude of any potential bias  Page: 14-15 Line: 276-295  Page: 15 Line: 297-308 |
| Interpretation | 20 | Give a cautious overall interpretation of results considering objectives, limitations, multiplicity of analyses, results from similar studies, and other relevant evidence  Page: 17 Line: 344-351 |
| Generalisability | 21 | Discuss the generalisability (external validity) of the study results  Page: 15 Line: 298-301 |
| Other information | | |
| Funding | 22 | Give the source of funding and the role of the funders for the present study and, if applicable, for the original study on which the present article is based  Funding information reported in additional information of the manuscript submission |

*Give information separately for exposed and unexposed groups.

**Note:** An Explanation and Elaboration article discusses each checklist item and gives methodological background and published examples of transparent reporting. The STROBE checklist is best used in conjunction with this article (freely available on the Web sites of PLoS Medicine at http://www.plosmedicine.org/, Annals of Internal Medicine at http://www.annals.org/, and Epidemiology at http://www.epidem.com/). Information on the STROBE Initiative is available at http://www.strobe-statement.org.
